# Global research trends in gut microbiota and cellular senescence: a bibliometric and visual analysis from 2015 to 2025

**DOI:** 10.3389/fmicb.2025.1623875

**Published:** 2025-08-06

**Authors:** Shaoyang Huang, Jiangyan Li, Dandan Gu, Zhengliang Li, Hongyun Chen, Wei Xiong

**Affiliations:** ^1^College of Basic Medical Sciences, Dali University, Dali, China; ^2^College of Life Sciences, Shaanxi Normal University, Xi’an, China; ^3^College of Clinical Medicine, Dali University, Dali, China; ^4^Department of Gastroenterology, Northeast Yunnan Regional Central Hospital, Zhaotong, China; ^5^College of Public Health, Dali University, Dali, China

**Keywords:** gut microbiota, cellular senescence, disease, CiteSpace, VOSviewer, bibliometric analysis

## Abstract

**Background:**

The human body’s intestinal microbiota is a vital “organ” that coexists with it and is intimately linked to both human health and illness. Human intestinal microbiota and its metabolites are a crucial component in the development of several diseases, according to an increasing number of studies that have started to examine the function of intestinal microbiota in various illnesses. Numerous recent studies have also shown a direct relationship between cellular senescence and the gut flora. The purpose of this study was to use bibliometric techniques to examine the themes and subjects of scholarly publications in this discipline during the past 10 or so years.

**Method:**

The Web of Science Core Collection (WOSCC) database was searched for material published between 2015 and 2025. The study used VOSviewer and Citespace to explore the characteristics of this literature. Specific analyzes covered the number of publications, countries/regions studied, research institutions, authors, journals, citations, and keyword hotspots.

**Results:**

This study analyzed 2,911 publications on research related to gut microbiota and cellular senescence between 2015 and 2025, with a rapid increase in annual publications from 2020 onwards, peaking in 2024 (492 publications), with the United States and China dominating in terms of publications, and the University of Groningen demonstrating excellent academic influence in this field, with Chen, Wei, De Vos, Paul and Zhang, Hao being the three most influential authors in this field, Nature is the most influential journal in its field with 5,857 total citations, “Oxidative stress,” “Alzheimer’s disease” and “immunotherapy” are current hot topics of research.

**Conclusion:**

Research in the field of gut microbiota and cellular aging is growing rapidly. Current research focuses on gut microbiota and disease mechanisms (e.g., Alzheimer’s disease, immunotherapy, oxidative stress) and clinical translation, and interdisciplinary collaborations and technological innovations are expected to drive further development in this field. This bibliometric study provides a comprehensive analysis of the field and offers new ideas for future research.

## 1 Introduction

The digestive system of humans constitutes a highly diverse and dynamic microecosystem, characterized by a complex interaction of various microbial populations. The human digestive system is home to over 100 trillion bacteria. These bacteria comprise roughly 1,000 to 7,000 distinct species, predominantly consisting of anaerobic, facultative anaerobic, and aerobic types. The gene repertoire encoded by the intestinal microbiota exceeds that of the human genome by more than 100-fold (Qin et al., 2010). This establishes a critical foundation for the microbiota’s involvement in diverse metabolic pathways and its pivotal role in maintaining human health. A plethora of studies have demonstrated that various human diseases, including obesity, diabetes, cardiovascular and cerebrovascular disorders, as well as autoimmune conditions, exhibit a significant correlation with alterations in the intestinal microbiota (Miyauchi et al., 2023; Ramirez-Macias et al., 2022). An optimal gut microbiome is integral in controlling metabolism, avoiding cancer and autoimmune diseases, reducing inflammation and infections, and preserving the brain-gut axis.

The postponement of aging has long been a fundamental aspiration for humanity, dating back to ancient civilizations. With the continuous advancements in medical technology and the rise in average life expectancy, the pursuit of anti-aging strategies has garnered significant attention. In order to achieve anti-aging, it is imperative to elucidate the underlying mechanisms responsible for the aging process. Traditionally, human life expectancy has been predominantly attributed to genetic factors. Environmental and lifestyle elements, such as nutrition and exercise, have played supplementary roles (Dato et al., 2017; Kondoh et al., 2020). Furthermore, the relationship between the symbiotic microorganisms residing within the human gut, collectively referred to as the gut microbiota, and the aging process has remained underexplored. From birth, the gut microbiota co-evolves with the host throughout the lifespan. It plays an important role in the immune system’s development and thus promotes overall health. Several studies have demonstrated that an aging gut microbiota is associated with intestinal inflammation and metabolic disturbances in the host (DeJong et al., 2020; Vemuri et al., 2018). As Nobel laureate Elie Metchnikoff proposed in 1907, aging is a consequence of the toxicity of specific products produced by the intestinal microbiota, and he posited that regulating the gut microbiota could potentially extend lifespan (Gasbarrini et al., 2016). In recent years, the pivotal role of the gut microbiota in the human aging process has garnered considerable attention. An expanding body of evidence increasingly supports the notion that the human gut microbiota plays an indispensable role in both health and disease. The structure, diversity, and functionality of the gut microbiota are progressively acknowledged as pivotal determinants in establishing and maintaining health, and are consequently often termed the “forgotten organ” (Blottiere et al., 2013). The cell serves as the fundamental unit of both the organism and the process of biological senescence. The concept of cellular senescence was initially introduced by Linskens et al. (1995) and defined as a physiological process predominantly characterized by the cessation of normal human cell growth and proliferation. Cellular senescence, a central mechanism in organismal aging, is intricately linked to the decline of tissue function and serves as a key driver of a variety of age-related illnesses, such as metabolic syndrome and neurodegenerative diseases, and chronic inflammation (Kang, 2019). A growing body of studies has demonstrated that dysregulation of the gut microbiota may expedite cellular senescence by triggering chronic inflammation, oxidative stress, and mitochondrial dysfunction (Chen et al., 2025; Ke et al., 2018; Soysal et al., 2020). Conversely, age-associated changes in the microenvironment may reshape the gut microbiota, establishing a complex network of bidirectional regulation (Tan et al., 2019).

Even with the large amount of research on gut microbiota and cellular senescence, little is known about the field’s general development trajectory, present condition, and emerging trends. In order to give researchers in the field unbiased, data-driven insights, this study aims to use bibliometric analysis to synthesize the relevant literature from the last few decades. It does this by using the WOSCC database, CiteSpace, and VOSviewer tools to map and analyze the major tendencies in this field of study.

## 2 Materials and methods

### 2.1 Data sources and search strategy

**Figure 1:**
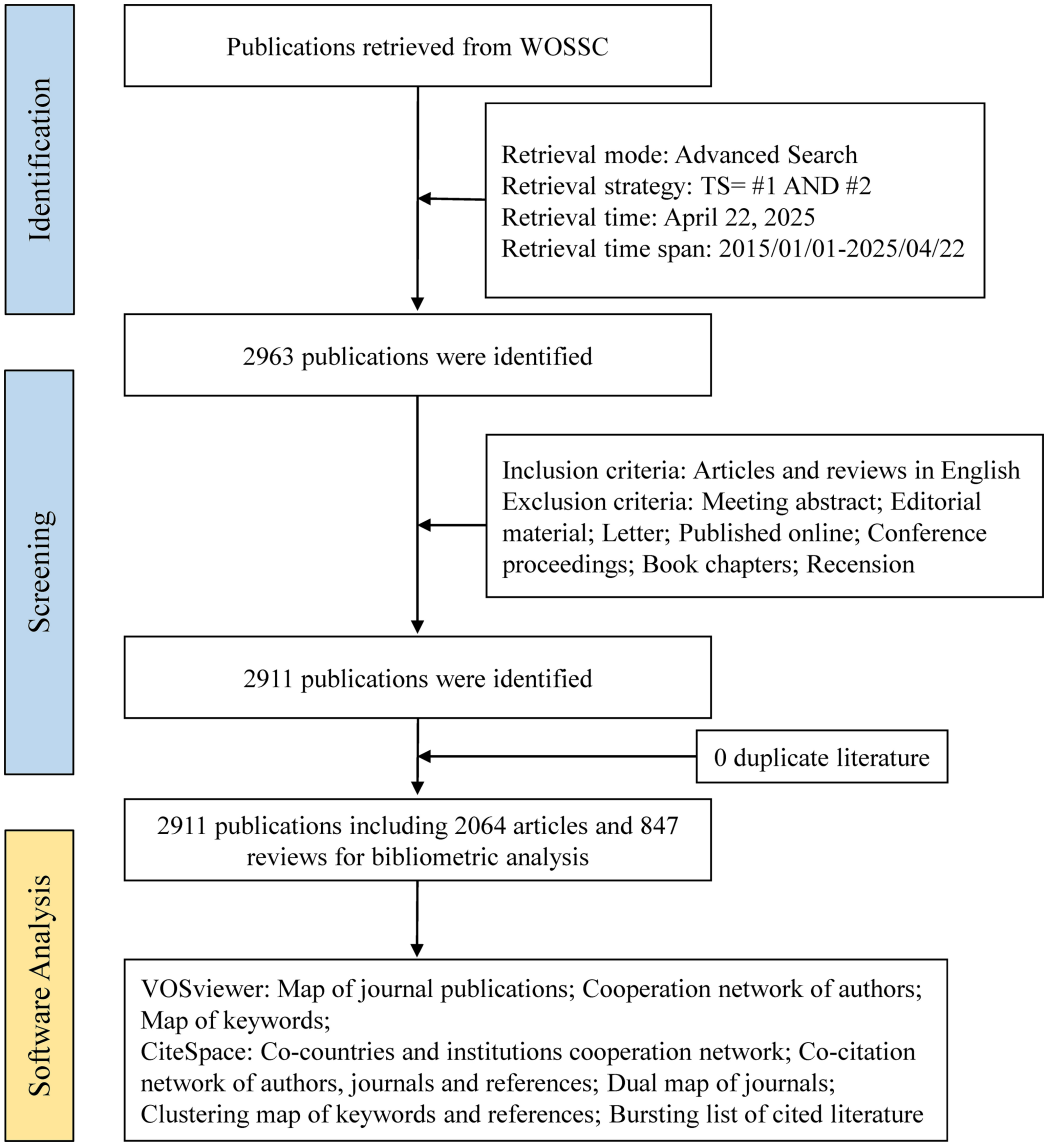
The flowchart of this study.

The Web of Science Core Collection (WoSCC) is one of the most widely utilized comprehensive academic databases globally, encompassing over 12,000 internationally recognized peer-reviewed journals across diverse fields such as engineering, medicine, the social sciences, the humanities, and the natural sciences (Chen et al., 2020). In this study, we conducted a systematic search of the WoSCC database for articles published between January 1, 2015, and April 22, 2025, using the primary subject terms “gut microbiome” and “cellular senescence,” along with associated keywords (detailed in Supplementary material 1). We selected this 10-year timeframe to ensure both the recency and representativeness of research trends in the field. A 10-year window is widely recognized in bibliometric studies as a balanced period that captures both emerging developments and established patterns, while maintaining data consistency and analytical depth. The literature retrieval was completed within a single day to ensure consistency and data integrity, and only English-language original research articles and review papers were included. Two independent researchers conducted the screening process. They excluded records unrelated to gut microbiota and cellular senescence research. Additionally, non-eligible publication types such as conference abstracts, case reports, letters, and preprints were excluded. Ultimately, 2,911 articles were included for analysis. The detailed screening workflow is illustrated in Figure 1.

### 2.2 Data analysis

Two bibliometric analysis tools were employed in this study: VOSviewer software version 1.6.19 (van Eck and Waltman, 2010) (developed by Leiden University, The Netherlands) and CiteSpace version 6.1. R6 software (developed by Drexel University, Philadelphia, Pennsylvania, United States) (Chen, 2004). Two distinct software programs that offer complementary capabilities and make it easier to visualize and analyze bibliometric and citation data in academic literature are VOSviewer and CitesSpace. Both tools are designed to help researchers analyze information more effectively by extracting relevant information from selected datasets and constructing visual images. Its parameter settings mainly include: data input, word processing, network construction and visualization settings, so as to achieve the purpose of visualization and analysis, we exported the downloaded literature data in text form, and then used 2 tools to identify the country, institution, journal and keywords related to the article and create co-occurrence diagrams, as well as extracting the article’s burst keywords, superimposing the journal’s bipartite diagrams and creating keyword timeline diagrams according to their frequency of occurrence to generate a keyword timeline graph, among other tasks.

## 3 Result

### 3.1 Annual publication volume and trend analysis

**Figure 2:**
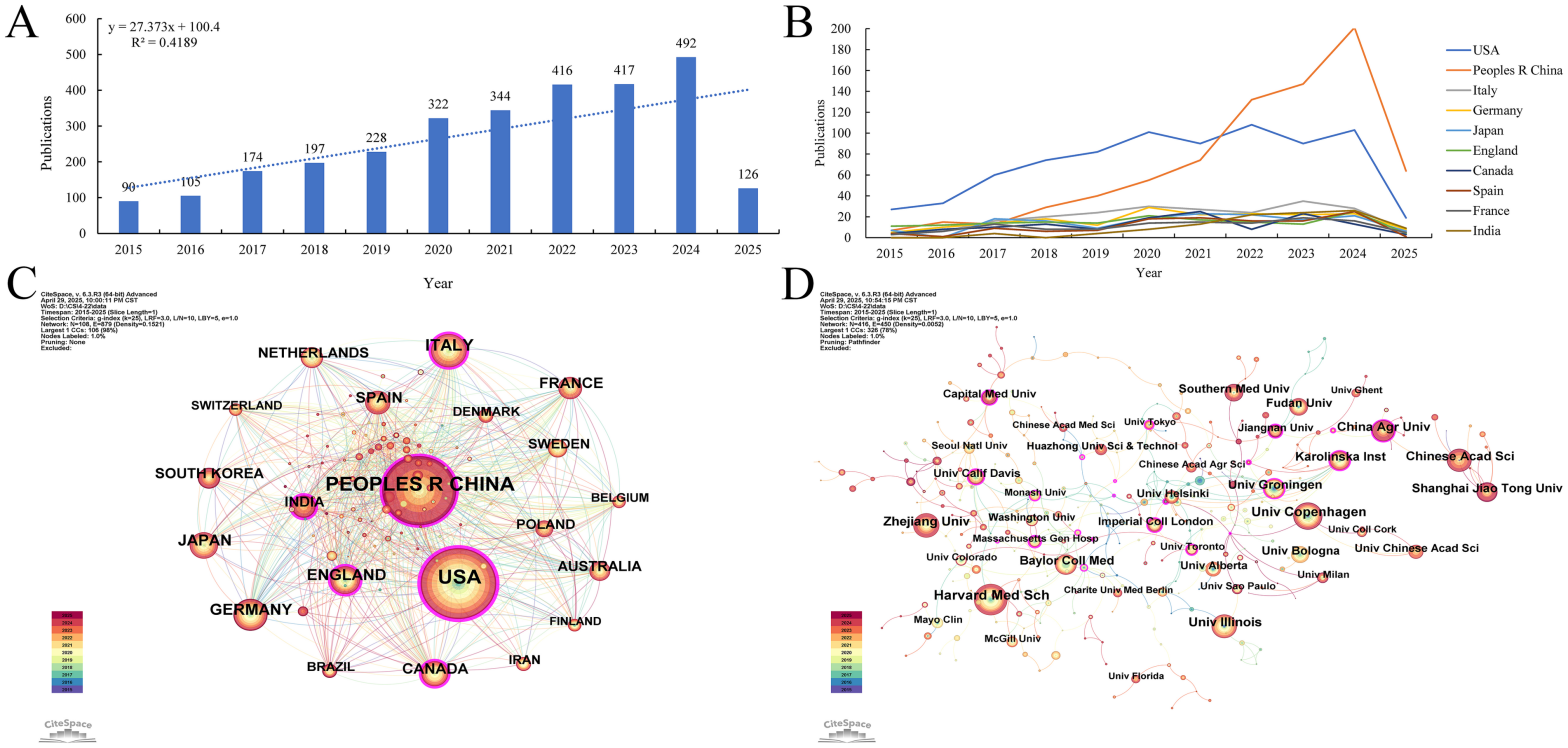
**(A)** Annualvolumeofpublications. **(B)** Line graph of national publications. **(C)** Networks of country cooperation. **(D)** Networks of institutional cooperation.

2,911 papers from 2015 to 2025 on gut microbiota and cellular senescence were carefully pulled from the Web of Science Core Collection for this study. The examination of publishing trends is depicted in Figure 2A. The results show a significant rise in research effort throughout the previous 10 years, indicating a strong and ongoing advancement in this area. In particular, starting in 2020, the number of publications each year has increased steadily, peaking at 492 pieces in 2024. This pattern highlights the ongoing scholarly interest in the mechanistic relationship between cellular senescence and gut microbiota, which has become a key area of study in intestinal microecology.

### 3.2 Analysis of country/institution publications and collaborations

This study further conducted a systematic analysis of the global distribution patterns of research on gut microbiota and cellular senescence. The results indicate that, as of 2025, a total of 108 countries and regions worldwide have engaged in related research. Annual publication trends from 2015 to 2025 for the top 10 publishing countries are depicted in Figure 2B. Regarding the volume of publications (Table 1), the United States (787 articles), China (777 articles), Italy (222 articles), Japan (164 articles), and the United Kingdom (161 articles) rank as the top five contributors globally. The United States and China demonstrate particular dominance. They account for 27.04 and 26.69% of total publications, respectively. Notably, China has exhibited a rapid upward trajectory in publication output, reflecting strong research momentum in this domain. In terms of academic impact, the United States leads the field with a total of 39,440 citations and an average of 50.11 citations per article. Its centrality index (0.38) also ranks first, highlighting its dominant position in this research area. In contrast, while China ranks second in total citations (15,584), its average of 20.06 citations per article suggests considerable room for improvement in academic influence. Analysis of the international collaboration network (Figure 2C) shows that the United States (0.38) and China (0.11) hold central positions and maintain close partnerships with countries such as Italy, the United Kingdom, Australia, Canada, Germany, and Spain. Such extensive international collaboration facilitates the production of high-impact research. It also strengthens the global research network in this domain.

**Table 1:** Statistics of top countries in terms of number of publications (top 10).

Rank	Country	Publications (2,911, %)	Centrality	Citations	Citation per publication
1	United States	787 (27.04%)	0.38	39,440	50.11
2	China	777 (26.69%)	0.11	15,584	20.06
3	Italy	222 (7.63%)	0.20	13,446	60.57
4	Germany	186 (6.39%)	0.07	10,799	58.06
5	Japan	164 (5.63%)	0.04	7,439	45.36
6	England	161 (5.53%)	0.12	12,372	76.84
7	Canada	134 (4.60%)	0.13	5,092	38.00
8	Spain	122 (4.19%)	0.09	7,691	63.04
9	France	121 (4.16%)	0.06	6,485	53.60
10	India	113 (3.88%)	0.14	4,536	40.14

Meanwhile, this study mapped the institutional collaboration network involving 4,127 research institutions worldwide from 2015 to 2025 (Figure 2D). In conjunction with the institutional publication scale (Table 2), this visualization illustrates the global distribution landscape of research on gut microbiota and cellular senescence. The analysis reveals a relatively balanced distribution of institutional output, with Harvard Medical School, the University of Copenhagen, Zhejiang University, and the University of Illinois ranking among the global leaders in relation to the volume of publications. Notably, the University of Groningen stands out for its academic impact, with 5,803 total citations and an average of 200.10 citations per article, indicating exceptional scholarly influence. Harvard Medical School and the University of Copenhagen are key hubs in the worldwide research network. Additional analysis shows they have established broad academic collaborations with institutions across various regions. Several regionally distinctive sub-clusters were identified, including a North America–Asia cluster led by Zhejiang University and the University of Illinois; a North America–Northern Europe cluster anchored by Baylor College of Medicine and Karolinska Institutet; and a Eurasian cluster centered on Shanghai Jiao Tong University, the University of Groningen, and the University of Bologna. These cross-regional and cross-institutional collaborative innovation networks have substantially contributed to advancing the field by facilitating the dissemination of scientific discoveries through academic exchange and resource sharing, thereby elevating the overall quality and global visibility of research on gut microbiota and cellular aging.

**Table 2:** Statistics on top institutions with the highest number of publications (top 10).

Rank	Institution	Publications (2,911, %)	Citations	Citation per publication
1	Harvard Medical University	45 (1.55%)	3,531	76.76
2	University Copenhagen	39 (1.34%)	942	23.55
3	Zhejiang University	35 (1.20%)	1,504	39.58
4	University Illinois	34 (1.17%)	686	19.60
5	Chinese Academy of Sciences	33 (1.13%)	591	17.38
6	China Agricultural University	30 (1.03%)	1,290	40.31
7	Baylor College of Medicine	29 (1.00%)	430	13.87
8	Shanghai Jiao Tong University	28 (0.96%)	982	31.68
9	Karolinska Institute	27 (0.93%)	2,490	85.86
10	University Groningen	27 (0.93%)	5,803	200.10

### 3.3 Analysis of journals and cited journals

**Figure 3:**
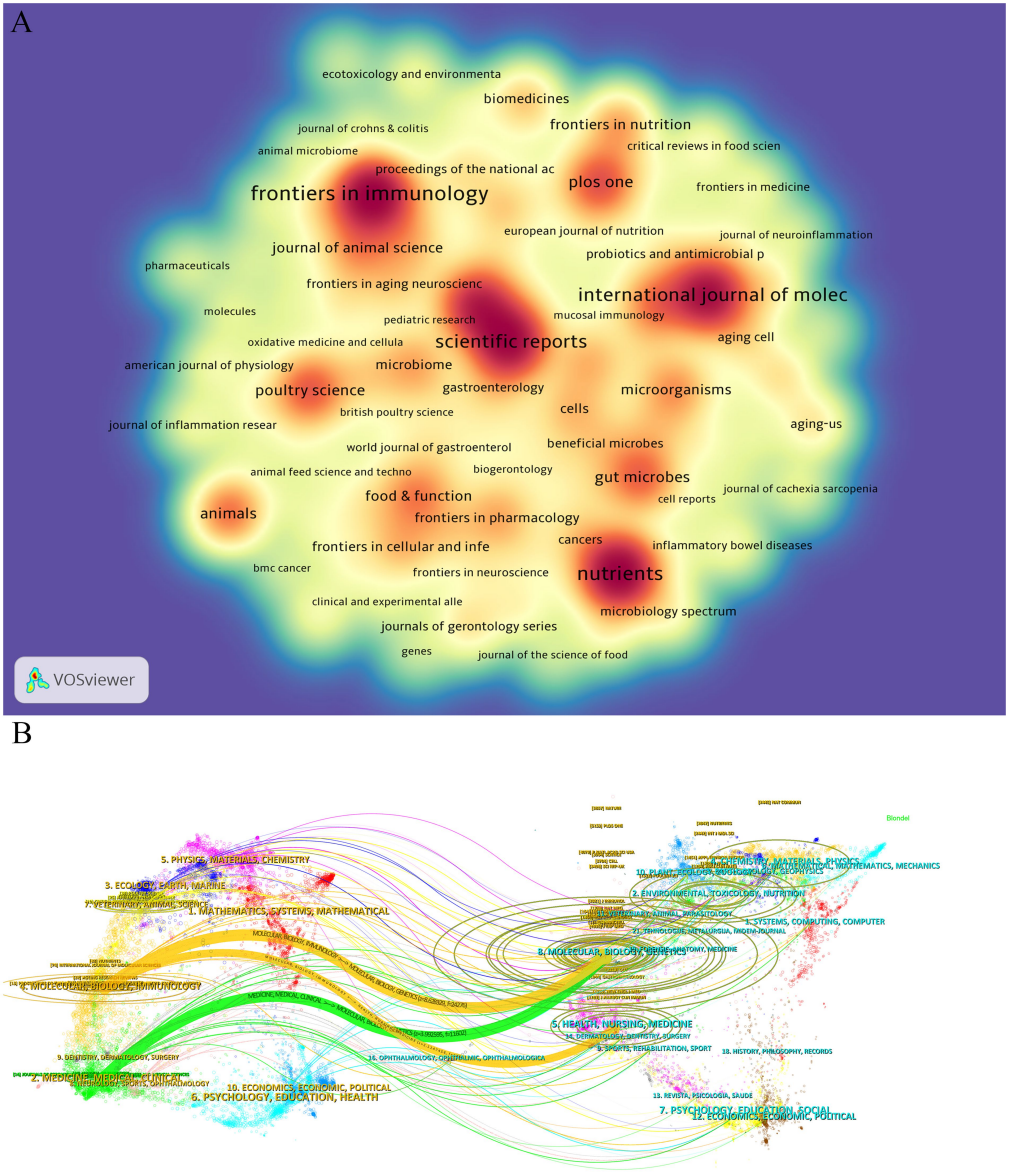
**(A)** Density map of journal publications. **(B)** Dual map of journals: the colored tracks represent citation connections, with citing journals on the left and cited journals on the right.

Based on the journal publication scale and density analysis (Table 3; Figure 3A), the data show that Frontiers in Immunology [Impact Factor (IF) = 5.7, Q1] ranks first with 89 publications, followed by Nutrients (IF = 4.8, Q1) and Scientific Reports (IF = 3.8, Q1). These journals collectively serve as core platforms for the dissemination of key findings in this field, suggesting that they are essential outlets for researchers to consider when selecting publication venues. As shown in (Table 4), Nature (IF = 50.5, Q1) leads in total citations with 5,857, followed by PLOS One (IF = 2.9, Q1) and PNAS (IF = 9.4, Q1), all of which are classified as Q1 journals in the Journal Citation Reports (JCR), underscoring their academic leadership in this domain.

**Table 3:** Statistics on top journals with the highest number of publications (top 10).

Rank	Journal	Publications (2,911, %)	Impact factor	Quartile in category
1	Frontiers in Immunology	89 (3.06%)	5.7	Q1
2	Nutrients	88 (3.02%)	4.8	Q1
3	Scientific Reports	77 (2.65%)	3.8	Q1
4	International Journal of Molecular Sciences	74 (2.54%)	4.9	Q1
5	Frontiers in Microbiology	60 (2.06%)	4.0	Q2
6	PLoS One	41 (1.41%)	2.9	Q1
7	POULTRY SCIENCE	38 (1.31%)	3.8	Q1
8	Animals	34 (1.17%)	2.7	Q1
9	Gut Microbes	34 (1.17%)	12.2	Q1
10	Ageing Research Reviews	32 (1.10%)	12.5	Q1

**Table 4:** Statistics of most cited journals (top 10).

Rank	Journal	Co-citations	Impact factor	Quartile in category
1	Nature	5,857	50.5	Q1
2	Plos One	5,153	2.9	Q1
3	PNAS	4,079	9.4	Q1
4	Science	3,964	44.8	Q1
5	Cell	3,704	45.6	Q1
6	Scientific Reports	3,498	3.8	Q1
7	Nutrients	2,847	4.8	Q1
8	Frontiers in Immunology	2,679	5.7	Q1
9	Nature Communications	2,445	14.7	Q1
10	International Journal of Molecular Sciences	2,442	4.9	Q1

To further explore disciplinary dynamics, a dual-map overlay analysis was conducted (Figure 3B) to examine the flow of scientific knowledge. The overlay displays two major citation pathways: one reveals that research published in journals classified under “molecular biology/genetics” is predominantly cited by journals in “molecular biology/immunology” and “clinical medicine”; the other shows that outputs from journals in “health care/medicine” are more frequently cited by journals in “molecular biology/immunology” than by those within their own field. This interdisciplinary citation structure highlights the dual nature of research on gut microbiota and cellular senescence, encompassing both fundamental biological inquiry and clinical translational relevance. These findings provide a theoretical framework for guiding researchers in selecting appropriate target journals for the dissemination of their work.

### 3.4 Analysis of authors and cited authors

**Figure 4:**
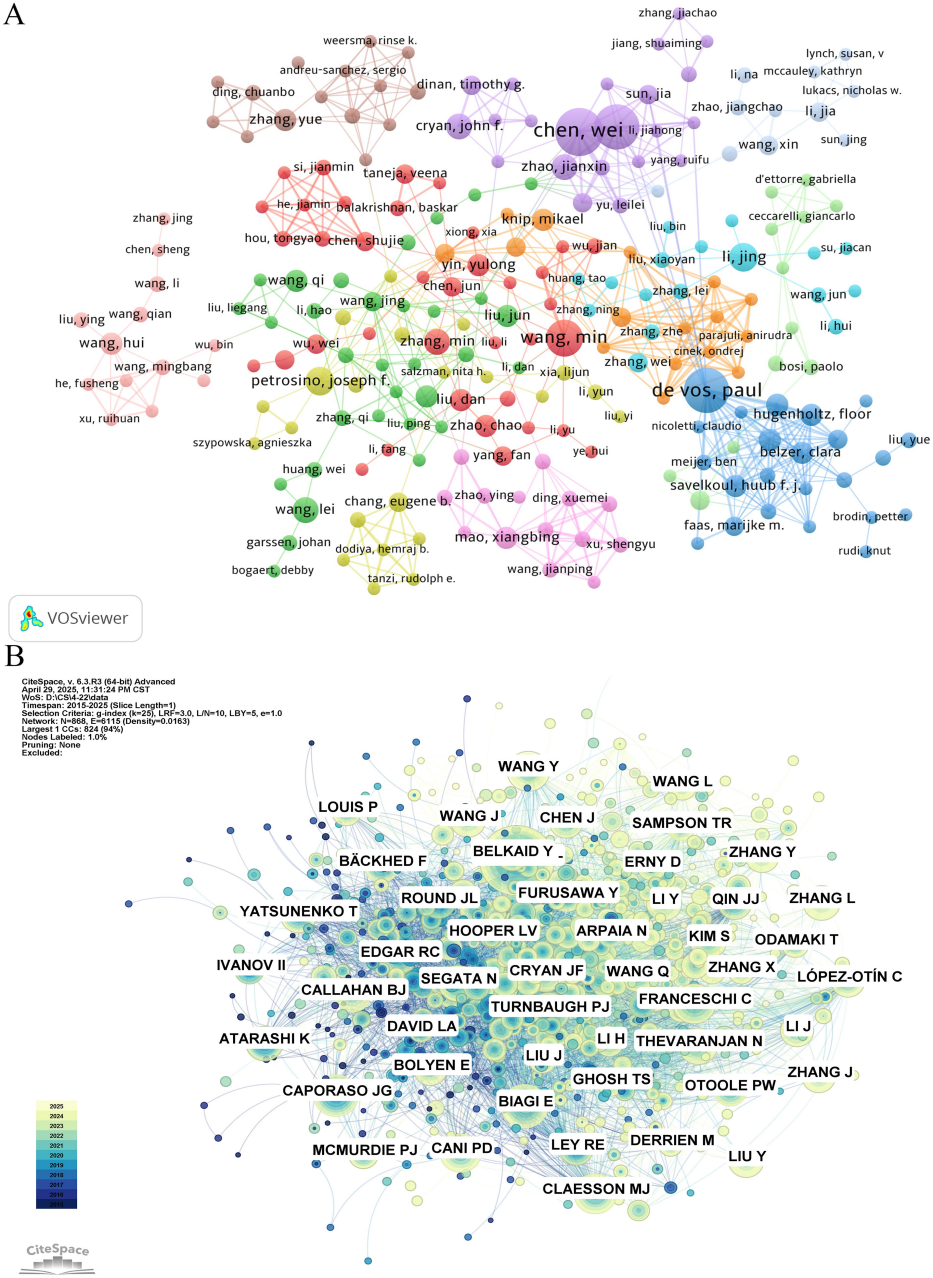
**(A)** Cooperation network of authors. **(B)** Cocitation network of authors.

The table of top published and cited authors (Table 5) shows the information of top published and cited authors in the field of gut microbiota and cellular senescence research. The results show that Chen, Wei (*n* = 15), De Vos, Paul (*n* = 14), and Zhang, Hao (*n* = 14) are the three authors with the highest number of publications in the field and have made outstanding contributions to the research in this area. In terms of academic impact, Biagi E (*n* = 241), Caporaso Jg (*n* = 215), and Claesson Mj (*n* = 209) are the three most highly cited scholars, whose research has had a profound impact in the field of intestinal microbiota and cellular aging research. (Figures 4A,B) presents the visualization results of the author collaboration network and cited relationship network. In the author cooperation network graph, the degree of collaboration between authors is shown by the thickness of the connecting line between nodes, and the size of the node is significantly connected with the frequency of writers’ appearances. Through the analysis, it is found that De Vos, Paul, is at the center of the research network and maintains close cooperation with many scholars. Notably, Wang, Min, Wang, Lei, Li, Jing, Petrosino, Joseph f., and Cryan, John f. play important leadership roles in their respective research teams.

**Table 5:** Top authors in terms of publications and citations (top 10).

Rank	Label	Documents	Rank	Label	Citations
1	Chen, Wei	15	1	Biagi E	241
2	De Vos, Paul	14	2	Caporaso Jg	215
3	Zhang, Hao	14	3	Claesson Mj	209
4	Wang, Min	11	4	Franceschi C	204
5	Li, Jing	8	5	Cani Pd	202
6	Petrosino, Joseph f.	8	6	Turnbaugh Pj	190
7	Cryan, John f.	7	7	Bäckhed F	156
8	Wang, Lei	7	8	Thevaranjan N	156
9	Zhang, Min	7	9	Wang Y	155
10	Zhao, Jianxin	7	10	Atarashi K	154

### 3.5 Keyword network analysis

**Figure 5:**
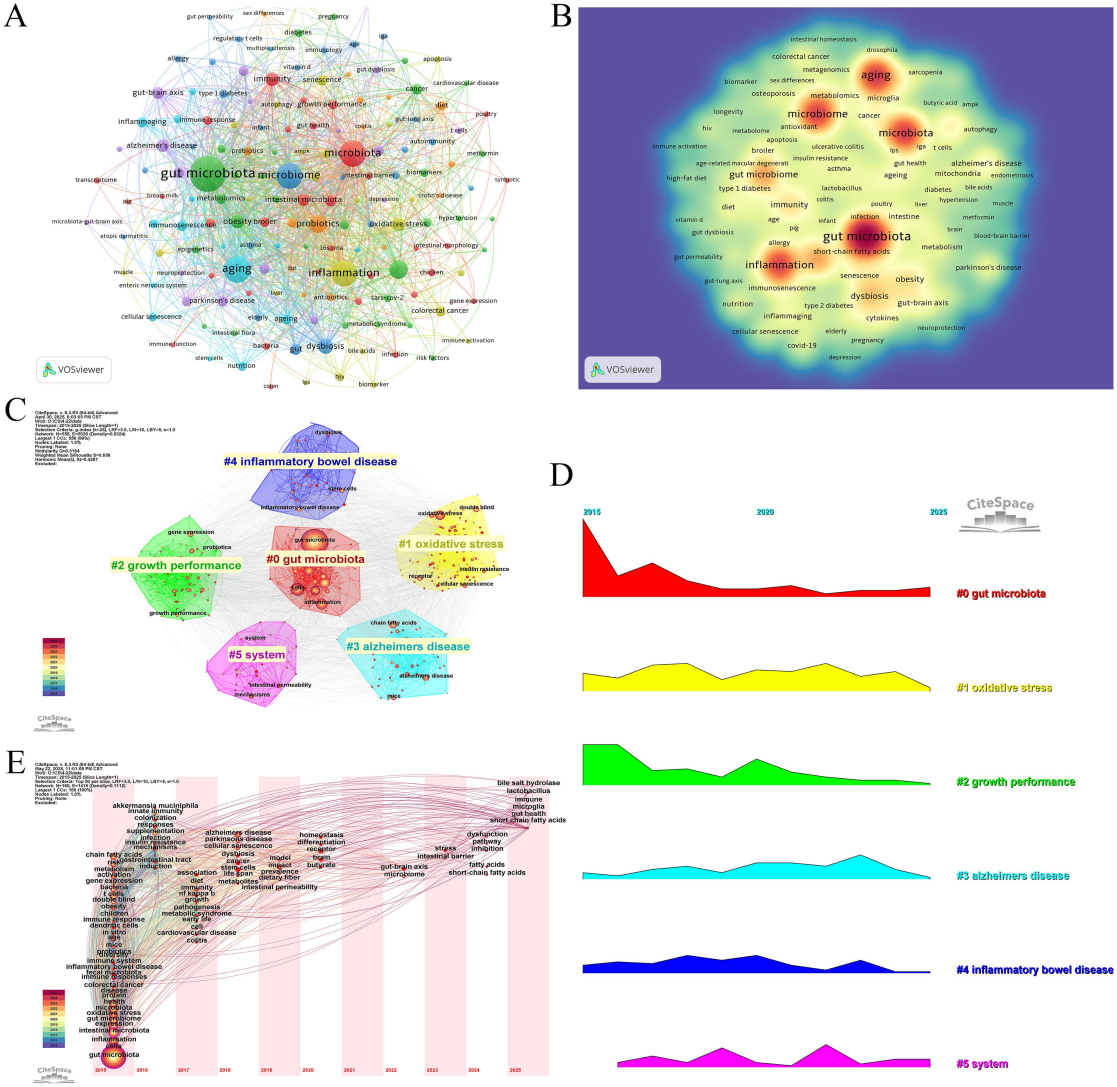
**(A)** Keywords co-occurrence frequency diagram. **(B)** Density map of keywords. **(C)** Clustering map of keywords. **(D)** Peak map of keyword clustering.**(E)** Burst Keyword Timeline Chart.

Keywords, as the core identifiers of academic papers, can accurately reflect research topics and disciplinary hotspots. In this study, the keywords in the research field of intestinal microbiota and cellular senescence were analyzed by co-occurrence network analysis and visual presentation using VOSviewer software (Figures 5A,B; Table 6). In the keyword co-occurrence network, the node size and color depth intuitively reflect the frequency of keywords and research heat. The analysis of high-frequency keywords showed that, in addition to the topic search terms

the most frequently occurring keywords were, in order, “cells” (358 times), “inflammation” (299 times), “intestinal microbiota” (286 times), ‘expression’ (279 times), and “oxidative stress” (253 times), these high-frequency words formed a strong semantic association with the research topic. Through the analysis of the co-occurrence network constructed by 160 keywords with frequency > 10 it was found that the network structure showed the characteristics of multi-center cross-distribution and the correlation between keywords was significant. In order to deeply analyze the hot trends of the research topic this study further generated keyword clustering views and peak maps (Figures 5C,D). Cluster analysis identified seven major research directions including “#0 gut microbiota” “#1 oxidative stress” “#2 growth performance” “#3 alzheimers disease” “#4 inflammatory bowel disease” “#5 system” “#6 bone matrix” a clustering structure that fully reflects the multidisciplinary nature of this research area. It is worth noting that “oxidative stress” shows obvious fluctuation of activity in the recent peaks suggesting that this direction has become one of the most promising research hotspots. By introducing a timeline change map of burst keywords (Figure 5E), it turned out that the earliest clusters of studies focused on “gut microbiota,” “inflammation,” “oxidative stress,” “gut flora,” and other foundational concepts (2015–2017), reflecting the initial mechanistic explorations. Subsequently, in 2018–2021, the research focus expanded into areas such as “Alzheimer’s disease,” “cellular senescence,” “intestinal permeability,” “butyric acid”, and other areas, indicating that the academic community is increasingly concerned with the study of neurodegenerative disease associations and metabolite-mediated mechanisms. Recent research hotspots from 2022 to 2025 include the “gut-brain axis”, “microglia”, “short-chain fatty acids”, “immunity”, and the “intestinal barrier”, reflecting a clear shift toward brain-gut interactions, immune regulation, and therapeutic applications. This trend highlights a transition from descriptive correlation studies to more refined mechanistic investigations and translational applications involving microbial metabolites, immune signaling pathways, and host barrier function.

**Table 6:** High-frequency keywords (top 20).

Rank	Label	Occurrences
1	Gut microbiota	1,164
2	Cells	358
3	Inflammation	299
4	Intestinal microbiota	286
5	Expression	279
6	Gut microbiome	255
7	Oxidative stress	253
8	Microbiota	248
9	Health	220
10	*t* cells	194
11	Chain fatty acids	183
12	Disease	178
13	Bacteria	160
14	Age	157
15	Risk	132
16	Metabolism	131
17	Activation	119
18	Alzheimers disease	116
19	Gene expression	116
20	Regulatory T cells	113

### 3.6 Literature co-citation analysis

**Figure 6:**
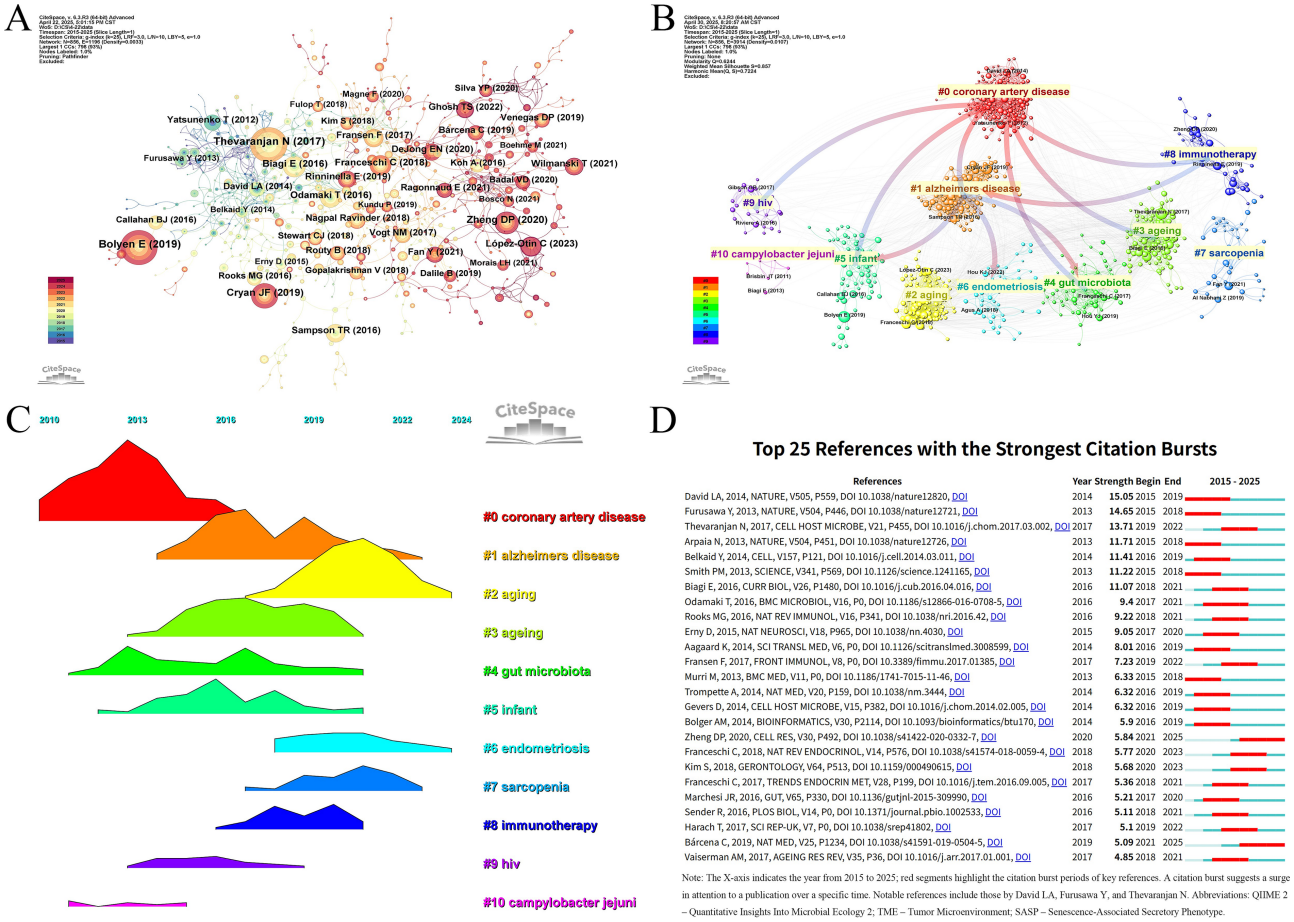
**(A)** Cocited network of literature. **(B)** Clustering of cocited literature. **(C)** Peak map of cocited literature. **(D)** Bursting list of cited literature.

In this study, a co-citation analysis of the literature network was conducted using CiteSpace to explore the high-impact literature as well as the co-occurrence relationship between the literature. As shown in (Figure 6A; Table 7), the article titled “Reproducible, interactive, scalable and extensible microbiome data science using QIIME 2” by Bolyen E et al., published in Nat Biotechnol, is a methodological paper introducing a widely used computational platform, but it does not present original biological data. The article by Thevaranjan N et al., published in Cell Host Microbe, is an original research paper that elucidates the mechanistic role of gut microbiota in aging. Meanwhile, Cryan JF et al.’s review article titled “The Microbiota-Gut-Brain Axis” in Physiol Rev. synthesizes prior findings to propose conceptual frameworks but does not contribute new experimental data. To improve transparency, we also examined the article types of the top 10 most co-cited publications in this field. Among them, 4 were review articles, 3 were original research papers, and 3 were methodological studies. Based on the literature co-citation network, we constructed two cluster maps (Figures 6B,C) to illustrate the distribution characteristics of research topics. The analysis revealed that “coronary artery disease” and “Alzheimer’s disease” formed the core research foundation, from which multiple research directions emerged, including “endometriosis,” “sarcopenia,” “immunotherapy,” and “*Campylobacter jejuni*,” among others. In particular, “#1 alzheimers disease” has formed a close network with “#0 coronary artery disease,” “#4 gut microbiota,” “#5 infant,” “#6 endometriosis,” and “#8 immunotherapy.” The research directions of “#5 infant,” “#6 endometriosis,” and “#8 immunotherapy” have formed a close network. From the perspective of chronological evolution, the research focus in the past 10 years or so has shown obvious stage characteristics. Early on, scholars focused on coronary artery disease (cluster 0). Subsequently, alzheimers disease (cluster 1), aging (cluster 3), gut microbiota (cluster 4), infant (cluster 5), and immunotherapy (cluster 8) have gradually become the hot research directions. Endometriosis (cluster 6) and sarcopenia (cluster 7) are current research hotspots that have attracted significant attention. (Figure 6D) shows that the research results of David LA, Furusawa Y, and Thevaranjan N published in NATURE and CELL HOST MICROBE, respectively, showed the strongest strength of emergence (*n* = 15.05, *n* = 14.65, and *n* = 13.71), and continued to lead the direction of academic development in recent years. Direction of academic development.

**Table 7:** Highly cocited literature (top 10).

Rank	Title	Journal	Author	Co-citations
1	Reproducible, interactive, scalable, and extensible microbiome data science using QIIME 2	Nature Biotechnology	Bolyen et al.,	90
2	Age-Associated Microbial Dysbiosis Promotes Intestinal Permeability, Systemic Inflammation, and Macrophage Dysfunction	Cell Host Microbe	Thevaranjan et al.,	90
3	The Microbiota-Gut-Brain Axis	Physiological Reviews	Cryan et al.,	65
4	Interaction between microbiota and immunity in health and disease	Cell Research	Zheng et al.,	58
5	Gut Microbiota and Extreme Longevity	Current Biology	Biagi et al.,	54
6	Inflammaging: a new immune-metabolic viewpoint for age-related diseases	Nature Reviews Endocrinology	Franceschi et al.,	52
7	Hallmarks of aging: An expanding universe	Cell	Lopez-Otin et al.,	51
8	Age-related changes in gut microbiota composition from newborn to centenarian: a cross-sectional study	BMC Microbiology	Odamaki et al.,	49
9	Aged Gut Microbiota Contributes to Systemical Inflammaging after Transfer to Germ-Free Mice	Frontiers in Immunology	Fransen et al.,	47
10	Gut Microbiota Regulate Motor Deficits and Neuroinflammation in a Model of Parkinson’s Disease	Cell	Sampson et al.,	47

## 4 Discussion

### 4.1 General information

Over the past 15 years, significant efforts have been dedicated to gut microbiota and cellular senescence research, leading to substantial progress. The rapid increase in publication output observed after 2020 in this study can largely be attributed to heightened scientific interest triggered by the COVID-19 pandemic. Supporting this assertion, keyword co-occurrence analysis revealed a significant increase in the usage frequency of terms such as “inflammatory response,” “immune response,” and “gut-brain axis” beginning in early 2020. For instance, Zuo et al. (2020) demonstrated substantial alterations in gut microbiota composition in COVID-19 patients, directly linking microbial dysbiosis to exacerbated inflammatory responses and increased disease severity. Similarly, Yeoh et al. (2021) reported significant microbiome disruptions correlated with COVID-19 severity, emphasizing the role of gut microbiota in regulating immune responses during viral infections. Furthermore, external databases such as the NIH Reporter and Dimensions.ai showed a marked increase in research funding specifically targeting microbiome and immune response studies following the pandemic, reflecting focused efforts to investigate microbiota-mediated immunity in the context of emerging infectious diseases (Prudencio and Costa, 2020). Collectively, these keyword trends and external funding data confirm that the COVID-19 pandemic acted as a critical catalyst. It drove a substantial increase in global research activities in gut microbiota and cellular senescence. Combined with the rapid advancement of high-throughput sequencing technologies (e.g., metagenomics, cost reductions in single-cell sequencing) (Jiang et al., 2021) and the application of artificial intelligence to analyze complex datasets (e.g., microbiome diversity, aging biomarkers) (Xiao et al., 2023), this has driven substantial growth in the field. Analysis of country and institutional contributions reveals that economically developed nations and their institutions significantly outpace developing countries in terms of publication volume and academic impact. This disparity may stem from insufficient research funding and a lack of critical technological infrastructure in developing countries. Developed countries with higher GDP are better positioned to fund research institutions and foster technological innovation, with researchers often opting for high-quality research platforms in these nations (Vasconcellos et al., 2018). Journal analysis indicates that the majority of publications in this field are concentrated in journals focusing on immunonutrition and molecular medicine, such as Frontiers in Immunology, Nutrients, and Scientific Reports. An encouraging trend is the increasing number of studies published in prestigious journals within the field, including Nature, PNAS, Science, and others. Notably, a 2020 report published in Nature suggests that the gut microbiota modulates inflammation onset in an amyotrophic lateral sclerosis mouse model, thereby delaying aging (Blacher et al., 2019), Additionally, a study published in PNAS proposes that methylglyoxal, a bacterial metabolite produced by the gut microbiota, may serve as a novel therapeutic target for delaying cellular senescence and associated pathophysiological processes (Shin et al., 2020); The publication of these studies has significantly enhanced the academic impact of the field. Author analysis reveals that Biagi E and Caporaso J. G. are the two most highly cited scholars, underscoring their substantial influence in this domain. Biagi E’s study analyzed the gut microbiota of semi-supercentenarians (105–109 years old) compared to adults, older adults, and centenarians. The study highlighted the potential of microbial interventions in delaying age-related diseases (Biagi et al., 2016), Caporaso et al.’s (2010) research focuses on microbial high-throughput sequencing. For instance, one of his studies emphasized the need to consider the impact of the gut microbiota in evaluating human development, nutritional requirements, physiological changes, and cellular senescence through microbiome sequencing. In addition, his recent systematic review assessed the degree of colonization and changes in the microbiome following fecal microbiota transplantation (FMT). This framework helps to more accurately quantify the reshaping effect of FMT on the microbiome and provides evaluation criteria and comparative basis for its clinical application in areas such as cellular aging (Herman et al., 2025). A closer examination of highly co-cited references reveals that these studies do more than shape the intellectual structure of the field; they also advance the mechanistic understanding of gut microbiota–cellular senescence interactions. For example, Thevaranjan N et al., (Cell Host and Microbe) published original research revealing how age-related microbial imbalances increase intestinal permeability and trigger systemic inflammation, thereby promoting macrophage dysfunction and cellular senescence through NF-κB-dependent pathways. In contrast, Cryan JF et al., (Physiological Reviews) and Franceschi C et al., (Nature Reviews Endocrinology) published comprehensive review articles on the ‘microbiota-gut-brain axis’ and ‘inflammatory aging,’ respectively. Although these review articles do not provide new experimental data, they contribute to a deeper understanding of the molecular interaction mechanisms underlying microbiota-driven aging processes, such as SASP secretion, mitochondrial dysfunction, and immune regulation.

### 4.2 Keyword hotspots and future trends

Keyword analysis is a useful technique for identifying the frontiers and emerging hotspots within a given research domain. Our analysis of existing studies reveals that the most frequently occurring keywords in this field include “gut microbiota,” “inflammation,” “intestinal microbiota,” “oxidative stress,” “Alzheimer’s disease,” and “age.” we combined visual analysis of co-cited references and their citation patterns. Our findings indicate that primary research hotspots in gut microbiota and cellular senescence focus on senescence-related diseases and underlying mechanisms. These will be discussed in detail below

#### 4.2.1 Alzheimer’s disease

The gut-brain axis constitutes a network of two-way communication that integrates brain and gastrointestinal functions (Dinan et al., 2014). According to recent research, the gut microbiota plays a critical role in the two-way communication between the gut and the brain and is essential to the functional dynamics of the gut-brain axis. Thus giving rise to the concept of the “microbial-gut-brain axis.” An increasing body of evidence underscores the significant role of the gut microbiota in regulating brain function and behavior (Qi et al., 2022). Among older adults, AD remains one of the most extensively studied age-related diseases, particularly in relation to alterations in the gut microbiota. The hallmark pathological features of AD encompass the accumulation of beta-amyloid plaques and the formation of neurofibrillary tangles (Xu et al., 2020). These pathological alterations are strongly associated with cellular senescence, a process that may be modulated by the activity of gut microbes (Bhat et al., 2012). A study by Sun et al. (2019) reported that elderly AD patient’s exhibit marked alterations in gut microbiota composition, which may increase intestinal permeability, induce inflammation, and subsequently trigger inflammatory cellular senescence, thereby heightening the risk of AD. Wu et al. (2017) demonstrated in a Drosophila AD model that intestinal bacterial infection promotes the accumulation of immune cells in the brain, triggering TNF-JNK-mediated neurodegeneration, which leads to CNS cell senescence and exacerbates AD progression. The cluster centered on “Alzheimer’s disease” emphasizes the role of the gut-brain axis in age-related neurodegeneration. Research in this cluster demonstrates that gut microbiota dysbiosis can increase intestinal permeability and systemic inflammation, which, in turn, promotes neuroinflammation and accelerates cellular senescence in brain tissues. This highlights a mechanistic link between microbial alterations, chronic inflammation, and the progression of neurodegenerative disorders.

#### 4.2.2 Sarcopenia

The gut-muscle axis describes the role of the gut microbiota in preserving muscle mass and physical function across the entire body (Ticinesi et al., 2019). The gut-muscle axis refers to the bidirectional interaction between gut microbiota and skeletal muscle, where microbial composition and metabolites influence muscle metabolism, inflammation, and functionality. Disruption of this axis with aging contributes to the development of sarcopenia, a condition marked by progressive loss of skeletal muscle mass, strength, and physical performance (Wang et al., 2024a). According to the European Working Group on Sarcopenia in Older People, sarcopenia is defined as “a syndrome characterized by limb dysfunction, diminished quality of life, and an increased risk of adverse events, including death, due to the progressive loss of skeletal muscle mass and strength” (Cruz-Jentoft et al., 2010). It is widely accepted that the loss of muscle mass in older adults is exacerbated by reduced appetite and food intake, which are associated with aging, thereby heightening the risk and severity of sarcopenia (Kurkcu et al., 2018). Recent years have seen increasing attention to the roles of gut microbiota and cellular senescence in myasthenia gravis research, with growing emphasis on their interactions. The prevailing theory posits that the gut microbiota significantly influences muscle mass and function by modulating the host’s inflammatory state, nutrient absorption, metabolic homeostasis, and immune responses (Nay et al., 2019). Imbalances in the intestinal microbiome can induce chronic low-grade inflammation, which accelerates cellular senescence. Senescent cells then secrete senescence-associated secretory phenotype (SASP) factors, further exacerbating the body’s inflammatory response and creating a vicious cycle (Sikora et al., 2011). Franceschi et al. (2000). In 2000, the first named this response the “inflammatory response-aging” phenomenon, and 2 recent studies have shown that serum inflammatory response factors (e.g., IL-10, IL-15, IL-6, etc.) correlate with sarcopenia, and that higher levels of systemic markers of inflammation seem to correlate with lower muscle strength and muscle mass (Pan et al., 2021; Tuttle et al., 2020). Changes in the gut microbiota can throw off the equilibrium between anti-infective and inflammatory reactions, accelerating cellular aging and causing damage to muscle tissue. The “sarcopenia” or “gut-muscle axis” cluster highlights how microbial changes with age can drive chronic low-grade inflammation and influence nutrient metabolism, ultimately contributing to muscle cell senescence and loss of muscle mass. This underscores the potential of targeting the gut microbiota to mitigate age-related muscle decline.

#### 4.2.3 Oxidative stress

Cells generate reactive oxygen species (ROS) as byproducts of bio-oxidative metabolism, which are typically neutralized by the body’s antioxidant defense mechanisms, thereby maintaining a dynamic equilibrium of ROS within the organism (Piccirillo et al., 2022). Several studies have established that gut microbiota may facilitate cellular senescence by exacerbating oxidative stress processes. A study by Mossad et al. (2022) compared the intestinal environments of young and old mice, revealing that gut microbiota dysbiosis resulted in elevated levels of N6-carboxymethyllysine metabolites, which promoted oxidative stress in aging microglia. This, in turn, led to mitochondrial dysfunction and directly contributed to microglial cell senescence. Yang et al. (2025) further demonstrated that the gut microbiota metabolite phenylacetylglutamine (PAGln) accelerates cellular senescence by activating the adrenergic receptor (ADR)-AMPK signaling pathway, which induces mitochondrial dysfunction and DNA damage, ultimately resulting in a marked increase in intracellular ROS levels. The “oxidative stress” cluster illustrates how gut microbiota imbalances contribute to increased production of ROS and mitochondrial dysfunction. This promotes DNA damage and the SASP, driving tissue aging. Studies in this area clarify the pivotal role of microbial metabolites and redox balance in modulating the pace of cellular aging.

#### 4.2.4 Immunotherapy

Immunotherapy represents one of the most significant breakthroughs in cancer research (Ager, 2023). Currently, the two primary immune checkpoint targets in clinical practice are cytotoxic T-lymphocyte-associated protein 4 (CTLA-4) and the programmed cell death-1/programmed death-ligand 1 (PD-1/PDL-1) axis, with related antibodies being employed in a range of cancer therapies. The PD-1/PDL-1 axis and its associated antibodies are integral to various cancer therapies (Arafat Hossain, 2024; Song et al., 2024). However, these drugs prove ineffective in the majority of patients. Recent research suggests that gut microbiota may interact with senescent cells to influence both tumors and the immune system, indicating that modulating gut microbiota could enhance the efficacy of immunotherapy. A previous study by Wang et al. (2024b) demonstrated that senescent cells can remodel the tumor microenvironment (TME) by secreting SASP factors, such as IL-6 and TGF-*β*, which inhibit CD8 + T-cell activity and facilitate immune escape. Conversely, a recent study by Cao et al. found that an intact gut microbiota can enhance CD8 + T-cell function and suppress tumor-promoting SPP1 + macrophages by activating the γδ T-cell-CD40L/CD40 axis. This finding implies that the detrimental effects of senescent cells may be partially mitigated by immune activation pathways modulated by specific microbiota. Moreover, the accumulation of pro-senescent metabolites, such as PAGln, may disrupt this balance, contributing to immunotherapy resistance (Cao et al., 2025). A study by Zhang et al. (2023) found that Clostridium nucleatum in the gut promotes SASP secretion in senescent esophageal cancer cells through the activation of the DNA damage response, thereby driving esophageal cancer progression and chemotherapy resistance. These findings underscore the potential of targeting the gut microbiota for esophageal cancer immunotherapy. These studies highlight the necessity for future research to explore the intricate colony-aging-immunity interaction network and to develop combination therapies that target the removal of tumor-promoting microbiota or the supplementation of immune-synergistic strains, aiming to overcome the efficacy limitations of current immune checkpoint inhibitors. Research grouped under “immunotherapy” explores how gut microbes and their metabolites modulate host immune responses, particularly in the tumor microenvironment. Key findings demonstrate that the gut microbiota can influence the effectiveness of immune checkpoint inhibitors by shaping the immune landscape, impacting both tumor progression and therapy resistance. The interplay between microbial metabolites, immune cell activation, and senescent cell SASP is critical for understanding and improving cancer immunotherapies.

#### 4.2.5 Biological mechanisms of intestinal microecological imbalance and host cell senescence

There is a complex and tight biological link between the gut microbiome and cellular senescence, which is currently recognized to involve the regulation of immune regulatory mechanisms, metabolic pathways, and SASPs. The gut microbiota plays a significant role in regulating host immunological homeostasis through immunomodulatory pathways. Dysbiosis leads to increased intestinal permeability, allowing endotoxins such as lipopolysaccharide (LPS) to enter the circulatory system and induce chronic low-grade inflammation (Zhang et al., 2022). This persistent inflammatory state activates signaling pathways such as NF-κB and p38 MAPK, driving cells into a state of senescence and exacerbating tissue damage and functional degeneration (Wang et al., 2020). Furthermore, certain good bacteria in the stomach, such as *Bifidobacterium* and *Lactobacillus*, can enhance regulatory T cell production and maintain Th17/Treg cell balance to inhibit inflammation-driven cellular senescence (Li et al., 2025); on the contrary, the expansion of pathogenic bacteria may promote immunosenescence, further aggravating tissue damage and functional degradation. immunosenescence, further accelerating the aging process of cells and tissues. From the perspective of metabolic pathways, gut microorganisms widely influence the aging process of host cells through their metabolites. Among them, short-chain fatty acids (SCFAs, such as acetic acid, propionic acid, and butyric acid) produced by fermentation of dietary fibers by intestinal flora have significant anti-inflammatory and anti-aging activities. Especially butyric acid is able to reduce oxidative stress and the expression of cell-cycle inhibitory factors, such as p16 and p21, through the inhibition of histone deacetylase (HDAC), activation of cell-protective mechanisms, such as the Nrf2 pathway, thus delaying cellular senescence (Lu et al., 2023; Saccon et al., 2021). Meanwhile, intestinal flora are also involved in the enterohepatic cycle of bile acids, which affects the host’s energy metabolism, redox homeostasis, and inflammatory response by regulating the activity of farnesol X receptor (FXR) and G-protein-coupled receptor (TGR5), and effectively reduces the expression level of SASP factors to alleviate cellular senescence. In addition, the metabolism of tryptophan by intestinal microorganisms generates indoles, which can activate the aromatic hydrocarbon receptor (AhR) of host cells, further inhibit the secretion of pro-inflammatory SASP factors, and alleviate the expression of cellular senescence (Lin et al., 2025). Gut microbes also significantly influence the body’s aging process by regulating cellular SASP. Healthy flora, especially butyric acid-producing flora, effectively reduce the release of SASP factors and protect tissues from accelerated senescence by reducing inflammatory signaling and inhibiting sustained activation of the NF-κB pathway. On the contrary, in the case of dysbiosis, harmful bacteria produce large amounts of pro-inflammatory metabolites, which significantly activate inflammatory signaling and promote the production of the SASP effect, further enhancing the senescence phenotype and accelerating the degradation of tissue function and the overall aging of the host (Abavisani et al., 2025; Liu et al., 2024).

In summary, given the growing body of research linking the gut microbiota to cellular senescence and age-related diseases, an important future direction is to translate these findings into practical therapeutic strategies. Promising interventions such as fecal microbiota transplantation (FMT), targeted probiotic therapies, and anti-inflammatory treatments are being increasingly explored. For example, a recent study (Zeng et al., 2023) found that FMT in young mice rejuvenated senescent hematopoietic stem cells in old mice by suppressing inflammation, thus providing new insights into therapeutic strategies for aging-associated hematological disorders. These findings suggest that fecal microbiota transplantation has demonstrated efficacy in restoring microbiota homeostasis and reducing systemic inflammation, offering a potential therapeutic tool for age-related conditions. Similarly, existing studies point to the possibility that individualized probiotic formulas tailored to age-specific microbial profiles may modulate key aging pathways such as mitochondrial function and oxidative stress responses (Gupta et al., 2024). Anti-inflammatory interventions targeting senescence-associated secretory phenotypes (SASPs), especially when combined with microbiota modulators, could create a synergistic approach to delaying or mitigating age-related diseases (Sharma et al., 2022). Future research should prioritize the validation of these interventions through clinical trials, the identification of microbiota-derived biomarkers of aging, and the integration of multi-omics data to develop precise and personalized anti-aging regimens. At the same time, we note that while interventions such as FMT and AI-assisted microbiome analysis are promising, there are several key challenges that require careful consideration. For example, FMT still faces significant limitations, including donor variability, risk of pathogen transmission, unknown long-term safety, and inconsistent efficacy in age-related applications (He et al., 2022). In addition, regulatory oversight of FMT remains fragmented, complicating its clinical standardization and large-scale application. Similarly, while artificial intelligence (AI) and machine learning have revolutionized the ability to analyze complex microbial datasets and predict aging phenotypes, their effectiveness relies heavily on the quality and representativeness of training data (Sudhakar et al., 2021). The “black box” nature of some AI models also raises concerns about interpretability and reproducibility, especially in clinical decision-making scenarios (Budhkar et al., 2025). The utility of AI-driven insights may be overestimated in the absence of robust validation on diverse populations and longitudinal datasets. Therefore, future research should address these limitations by improving FMT donor screening protocols, standardizing treatment frameworks, and developing transparent clinically interpretable AI models with strong generalization capabilities.

## 5 Conclusion

In conclusion, although this study suffers from some limitations inherent to bibliometric studies. For instance, our analysis primarily relies on bibliometric data, focusing on paper counts and citation frequencies, which does not provide a comprehensive evaluation of study quality. Additionally, the data used in this study were exclusively sourced from the WOSCC, potentially overlooking relevant literature from other databases, particularly non-English language publications, which could result in biased findings. Nevertheless, this study offers valuable insights into the current status and frontiers of research on gut microbiota and cellular senescence from 2015 to 2025, while suggesting potential avenues for future exploration. We strongly hope that this work will inspire further scholarly discussions and foster stronger collaborations between researchers and institutions to address the existing gaps in this field.

## Data Availability

The original contributions presented in the study are included in the article/Supplementary material, further inquiries can be directed to the corresponding author/s.

## References

[ref1] Abavisani M. Faraji S. Ebadpour N. Karav S. Sahebkar A. (2025). Beyond the Hayflick limit: how microbes influence cellular aging. Ageing Res. Rev. 104:102657. doi: 10.1016/j.arr.2025.102657, PMID: 39788433

[ref2] Ager A. (2023). Cancer immunotherapy: T cells and neutrophils working together to attack cancers. Cell 186, 1304–1306. doi: 10.1016/j.cell.2023.03.005, PMID: 37001496

[ref3] Arafat Hossain M. (2024). A comprehensive review of immune checkpoint inhibitors for cancer treatment. Int. Immunopharmacol. 143:113365. doi: 10.1016/j.intimp.2024.113365, PMID: 39447408

[ref4] Bhat R. Crowe E. P. Bitto A. Moh M. Katsetos C. D. Garcia F. U. . (2012). Astrocyte senescence as a component of Alzheimer's disease. PLoS One 7:e45069. doi: 10.1371/journal.pone.0045069 22984612 PMC3440417

[ref5] Biagi E. Franceschi C. Rampelli S. Severgnini M. Ostan R. Turroni S. . (2016). Gut microbiota and extreme longevity. Curr. Biol. 26, 1480–1485. doi: 10.1016/j.cub.2016.04.016 27185560

[ref6] Blacher E. Bashiardes S. Shapiro H. Rothschild D. Mor U. Dori-Bachash M. . (2019). Potential roles of gut microbiome and metabolites in modulating ALS in mice. Nature 572, 474–480. doi: 10.1038/s41586-019-1443-5, PMID: 31330533

[ref7] Blottiere H. M. de Vos W. M. Ehrlich S. D. Dore J. (2013). Human intestinal metagenomics: state of the art and future. Curr. Opin. Microbiol. 16, 232–239. doi: 10.1016/j.mib.2013.06.006, PMID: 23870802

[ref8] Budhkar A. Song Q. Su J. Zhang X. (2025). Demystifying the black box: A survey on explainable artificial intelligence (XAI) in bioinformatics. Comput. Struct. Biotechnol. J. 27, 346–359. doi: 10.1016/j.csbj.2024.12.027, PMID: 39897059 PMC11782883

[ref9] Cao M. Deng Y. Hao Q. Yan H. Wang Q. L. Dong C. . (2025). Single-cell transcriptomic analysis reveals gut microbiota-immunotherapy synergy through modulating tumor microenvironment. Signal Transduct. Target. Ther. 10:140. doi: 10.1038/s41392-025-02226-7, PMID: 40312419 PMC12045981

[ref10] Caporaso J. G. Kuczynski J. Stombaugh J. Bittinger K. Bushman F. D. Costello E. K. . (2010). QIIME allows analysis of high-throughput community sequencing data. Nat. Methods 7, 335–336. doi: 10.1038/nmeth.f.303, PMID: 20383131 PMC3156573

[ref11] Chen C. (2004). Searching for intellectual turning points: progressive knowledge domain visualization. Proc. Natl. Acad. Sci. USA 101, 5303–5310. doi: 10.1073/pnas.0307513100, PMID: 14724295 PMC387312

[ref12] Chen S. Lu Q. Bai J. Deng C. Wang Y. Zhao Y. (2020). Global publications on stigma between 1998-2018: A bibliometric analysis. J. Affect. Disord. 274, 363–371. doi: 10.1016/j.jad.2020.05.006, PMID: 32469828

[ref13] Chen W. Zou H. Xu H. Cao R. Zhang Y. Ma Y. . (2025). Exploring the mechanisms of testicular aging: advances in biomarker research. Aging Dis.:01. doi: 10.14336/AD.2025.0070, PMID: 40153586 PMC12834421

[ref14] Cruz-Jentoft A. J. Baeyens J. P. Bauer J. M. Boirie Y. Cederholm T. Landi F. . (2010). Sarcopenia: European consensus on definition and diagnosis: report of the European working group on sarcopenia in older people. Age Ageing 39, 412–423. doi: 10.1093/ageing/afq034, PMID: 20392703 PMC2886201

[ref15] Dato S. Rose G. Crocco P. Monti D. Garagnani P. Franceschi C. . (2017). The genetics of human longevity: an intricacy of genes, environment, culture and microbiome. Mech. Ageing Dev. 165, 147–155. doi: 10.1016/j.mad.2017.03.011, PMID: 28390822

[ref16] DeJong E. N. Surette M. G. Bowdish D. M. E. (2020). The gut microbiota and unhealthy aging: disentangling cause from consequence. Cell Host Microbe 28, 180–189. doi: 10.1016/j.chom.2020.07.013, PMID: 32791111

[ref17] Dinan T. G. Borre Y. E. Cryan J. F. (2014). Genomics of schizophrenia: time to consider the gut microbiome? Mol. Psychiatry 19, 1252–1257. doi: 10.1038/mp.2014.93, PMID: 25288135

[ref18] Franceschi C. Bonafe M. Valensin S. Olivieri F. De Luca M. Ottaviani E. . (2000). Inflamm-aging. An evolutionary perspective on immunosenescence. Ann. N. Y. Acad. Sci. 908, 244–254. doi: 10.1111/j.1749-6632.2000.tb06651.x 10911963

[ref19] Gasbarrini G. Bonvicini F. Gramenzi A. (2016). Probiotics history. J. Clin. Gastroenterol. 50, S116–S119. doi: 10.1097/MCG.0000000000000697 27741152

[ref20] Gupta N. El-Gawaad N. S. A. Mallasiy L. O. Gupta H. Yadav V. K. Alghamdi S. . (2024). Microbial dysbiosis and the aging process: a review on the potential age-deceleration role of *Lactiplantibacillus plantarum*. Front. Microbiol. 15:1260793. doi: 10.3389/fmicb.2024.1260793, PMID: 38440135 PMC10909992

[ref21] He R. Li P. Wang J. Cui B. Zhang F. Zhao F. (2022). The interplay of gut microbiota between donors and recipients determines the efficacy of fecal microbiota transplantation. Gut Microbes 14:2100197. doi: 10.1080/19490976.2022.2100197, PMID: 35854629 PMC9302524

[ref22] Herman C. Barker B. M. Bartelli T. F. Chandra V. Krajmalnik-Brown R. Jewell M. . (2025). A review of transplantation assessment following faecal microbiota transplantation. doi: 10.1080/19490976.2025.2525478, PMID: 40605266 PMC12233830

[ref23] Jiang X. Pan W. Chen M. Wang W. Song W. Lin G. N. (2021). Integrative enrichment analysis of gene expression based on an artificial neuron. BMC Med. Genet. 14:173. doi: 10.1186/s12920-021-00988-x, PMID: 34433483 PMC8386081

[ref24] Kang C. (2019). Senolytics and Senostatics: A two-pronged approach to target cellular senescence for delaying aging and age-related diseases. Mol. Cells 42, 821–827. doi: 10.14348/molcells.2019.0298, PMID: 31838837 PMC6939651

[ref25] Ke Y. Li D. Zhao M. Liu C. Liu J. Zeng A. . (2018). Gut flora-dependent metabolite trimethylamine-N-oxide accelerates endothelial cell senescence and vascular aging through oxidative stress. Free Radic. Biol. Med. 116, 88–100. doi: 10.1016/j.freeradbiomed.2018.01.007, PMID: 29325896

[ref26] Kondoh H. Kameda M. Yanagida M. (2020). Whole blood metabolomics in aging research. Int. J. Mol. Sci. 22:175. doi: 10.3390/ijms22010175, PMID: 33375345 PMC7796096

[ref27] Kurkcu M. Meijer R. I. Lonterman S. Muller M. de van der Schueren M. A. E. (2018). The association between nutritional status and frailty characteristics among geriatric outpatients. Clin Nutr ESPEN 23, 112–116. doi: 10.1016/j.clnesp.2017.11.006, PMID: 29460785

[ref28] Li A. Kou R. Wang R. Wang J. Zhang B. Liu J. . (2025). 2'-Fucosyllactose attenuates aging-related metabolic disorders through modulating gut microbiome-T cell axis. Aging Cell 24:e14343. doi: 10.1111/acel.14343 39301860 PMC11709090

[ref29] Lin X. Xia L. Zhou Y. Xie J. Tuo Q. Lin L. . (2025). Crosstalk between bile acids and intestinal epithelium: multidimensional roles of Farnesoid X receptor and Takeda G protein receptor 5. Int. J. Mol. Sci. 26:240. doi: 10.3390/ijms26094240, PMID: 40362481 PMC12072030

[ref30] Linskens M. H. Harley C. B. West M. D. Campisi J. Hayflick L. (1995). Replicative senescence and cell death. Science 267:17. doi: 10.1126/science.7848496 7848496

[ref31] Liu S. Hu H. Zhang M. Zhang Y. Geng R. Jin Y. . (2024). Puerarin delays mammary gland aging by regulating gut microbiota and inhibiting the p38MAPK signaling pathway. J. Agric. Food Chem. 72, 10879–10896. doi: 10.1021/acs.jafc.3c09444, PMID: 38686994

[ref32] Lu X. Li J. Ma Y. Khan I. Yang Y. Li Y. . (2023). Fermented Angelica sinensis activates Nrf 2 signaling and modulates the gut microbiota composition and metabolism to attenuate D-gal induced liver aging. Food Funct. 14, 215–230. doi: 10.1039/d2fo01637k, PMID: 36477974

[ref33] Miyauchi E. Shimokawa C. Steimle A. Desai M. S. Ohno H. (2023). The impact of the gut microbiome on extra-intestinal autoimmune diseases. Nat. Rev. Immunol. 23, 9–23. doi: 10.1038/s41577-022-00727-y, PMID: 35534624

[ref34] Mossad O. Batut B. Yilmaz B. Dokalis N. Mezo C. Nent E. . (2022). Gut microbiota drives age-related oxidative stress and mitochondrial damage in microglia via the metabolite N (6)-carboxymethyllysine. Nat. Neurosci. 25, 295–305. doi: 10.1038/s41593-022-01027-3, PMID: 35241804

[ref35] Nay K. Jollet M. Goustard B. Baati N. Vernus B. Pontones M. . (2019). Gut bacteria are critical for optimal muscle function: a potential link with glucose homeostasis. Am. J. Physiol. Endocrinol. Metab. 317, E158–E171. doi: 10.1152/ajpendo.00521.2018, PMID: 31039010

[ref36] Pan L. Xie W. Fu X. Lu W. Jin H. Lai J. . (2021). Inflammation and sarcopenia: A focus on circulating inflammatory cytokines. Exp. Gerontol. 154:111544. doi: 10.1016/j.exger.2021.111544, PMID: 34478826

[ref37] Piccirillo S. Magi S. Preziuso A. Serfilippi T. Cerqueni G. Orciani M. . (2022). The hidden notes of redox balance in neurodegenerative diseases. Antioxidants (Basel) 11:1456. doi: 10.3390/antiox11081456, PMID: 35892658 PMC9331713

[ref38] Prudencio M. Costa J. C. (2020). Research funding after COVID-19. Nat. Microbiol. 5:986. doi: 10.1038/s41564-020-0768-z, PMID: 32710091

[ref39] Qi M. Tan B. Wang J. Liao S. Deng Y. Ji P. . (2022). The microbiota-gut-brain axis: A novel nutritional therapeutic target for growth retardation. Crit. Rev. Food Sci. Nutr. 62, 4867–4892. doi: 10.1080/10408398.2021.1879004, PMID: 33523720

[ref40] Qin J. Li R. Raes J. Arumugam M. Burgdorf K. S. Manichanh C. . (2010). A human gut microbial gene catalogue established by metagenomic sequencing. Nature 464, 59–65. doi: 10.1038/nature08821, PMID: 20203603 PMC3779803

[ref41] Ramirez-Macias I. Orenes-Pinero E. Camelo-Castillo A. Rivera-Caravaca J. M. Lopez-Garcia C. Marin F. (2022). Novel insights in the relationship of gut microbiota and coronary artery diseases. Crit. Rev. Food Sci. Nutr. 62, 3738–3750. doi: 10.1080/10408398.2020.1868397, PMID: 33399007

[ref42] Saccon T. D. Nagpal R. Yadav H. Cavalcante M. B. Nunes A. D. C. Schneider A. . (2021). Senolytic combination of Dasatinib and quercetin alleviates intestinal senescence and inflammation and modulates the gut microbiome in aged mice. J. Gerontol. A Biol. Sci. Med. Sci. 76, 1895–1905. doi: 10.1093/gerona/glab002 33406219 PMC8514064

[ref43] Sharma R. Diwan B. Sharma A. Witkowski J. M. (2022). Emerging cellular senescence-centric understanding of immunological aging and its potential modulation through dietary bioactive components. Biogerontology 23, 699–729. doi: 10.1007/s10522-022-09995-6, PMID: 36261747 PMC9581456

[ref44] Shin M. G. Lee J. W. Han J. S. Lee B. Jeong J. H. Park S. H. . (2020). Bacteria-derived metabolite, methylglyoxal, modulates the longevity of C. elegans through TORC2/SGK-1/DAF-16 signaling. Proc. Natl. Acad. Sci. USA 117, 17142–17150. doi: 10.1073/pnas.1915719117, PMID: 32636256 PMC7382248

[ref45] Sikora E. Arendt T. Bennett M. Narita M. (2011). Impact of cellular senescence signature on ageing research. Ageing Res. Rev. 10, 146–152. doi: 10.1016/j.arr.2010.10.002, PMID: 20946972

[ref46] Song D. Hou S. Ma N. Yan B. Gao J. (2024). Efficacy and safety of PD-1/PD-L1 and CTLA-4 immune checkpoint inhibitors in the treatment of advanced colorectal cancer: a systematic review and meta-analysis. Front. Immunol. 15:1485303. doi: 10.3389/fimmu.2024.1485303, PMID: 39555073 PMC11563947

[ref47] Soysal P. Arik F. Smith L. Jackson S. E. Isik A. T. (2020). Inflammation, frailty and cardiovascular disease. Adv. Exp. Med. Biol. 1216, 55–64. doi: 10.1007/978-3-030-33330-0_7, PMID: 31894547

[ref48] Sudhakar P. Machiels K. Verstockt B. Korcsmaros T. Vermeire S. (2021). Computational biology and machine learning approaches to understand mechanistic microbiome-host interactions. Front. Microbiol. 12:618856. doi: 10.3389/fmicb.2021.618856, PMID: 34046017 PMC8148342

[ref49] Sun J. Liu S. Ling Z. Wang F. Ling Y. Gong T. . (2019). Fructooligosaccharides ameliorating cognitive deficits and neurodegeneration in APP/PS1 transgenic mice through modulating gut microbiota. J. Agric. Food Chem. 67, 3006–3017. doi: 10.1021/acs.jafc.8b07313, PMID: 30816709

[ref50] Tan Y. Wei Z. Chen J. An J. Li M. Zhou L. . (2019). Save your gut save your age: the role of the microbiome in stem cell ageing. J. Cell. Mol. Med. 23, 4866–4875. doi: 10.1111/jcmm.14373, PMID: 31207055 PMC6653314

[ref51] Ticinesi A. Nouvenne A. Cerundolo N. Catania P. Prati B. Tana C. . (2019). Gut microbiota, muscle mass and function in aging: A focus on physical frailty and sarcopenia. Nutrients 11:1633. doi: 10.3390/nu11071633, PMID: 31319564 PMC6683074

[ref52] Tuttle C. S. L. Thang L. A. N. Maier A. B. (2020). Markers of inflammation and their association with muscle strength and mass: A systematic review and meta-analysis. Ageing Res. Rev. 64:101185. doi: 10.1016/j.arr.2020.101185 32992047

[ref53] van Eck N. J. Waltman L. (2010). Software survey: VOSviewer, a computer program for bibliometric mapping. Scientometrics 84, 523–538. doi: 10.1007/s11192-009-0146-3, PMID: 20585380 PMC2883932

[ref54] Vasconcellos A. G. Fonseca E. F. B. P. Morel C. M. (2018). Revisiting the concept of innovative developing countries (IDCs) for its relevance to health innovation and neglected tropical diseases and for the prevention and control of epidemics. PLoS Negl. Trop. Dis. 12:e0006469. doi: 10.1371/journal.pntd.0006469 30001318 PMC6042684

[ref55] Vemuri R. Gundamaraju R. Shastri M. D. Shukla S. D. Kalpurath K. Ball M. . (2018). Gut microbial changes, interactions, and their implications on human lifecycle: An ageing perspective. Biomed. Res. Int. 2018, 1–13. doi: 10.1155/2018/4178607, PMID: 29682542 PMC5846367

[ref56] Wang S. Ahmadi S. Nagpal R. Jain S. Mishra S. P. Kavanagh K. . (2020). Lipoteichoic acid from the cell wall of a heat killed *Lactobacillus paracasei* D3-5 ameliorates aging-related leaky gut, inflammation and improves physical and cognitive functions: from *C. elegans* to mice. Geroscience 42, 333–352. doi: 10.1007/s11357-019-00137-4, PMID: 31814084 PMC7031475

[ref57] Wang Z. Chen Y. Fang H. Xiao K. Wu Z. Xie X. . (2024b). Reprogramming cellular senescence in the tumor microenvironment augments cancer immunotherapy through multifunctional nanocrystals. Sci. Adv. 10:eadp7022. doi: 10.1126/sciadv.adp7022, PMID: 39485841 PMC11529718

[ref58] Wang M. Ren F. Zhou Y. He Y. Du T. Tan Y. (2024a). Age-related sarcopenia and altered gut microbiota: A systematic review. Microb. Pathog. 195:106850. doi: 10.1016/j.micpath.2024.106850, PMID: 39142365

[ref59] Wu S. C. Cao Z. S. Chang K. M. Juang J. L. (2017). Intestinal microbial dysbiosis aggravates the progression of Alzheimer's disease in Drosophila. Nat. Commun. 8:24. doi: 10.1038/s41467-017-00040-6, PMID: 28634323 PMC5478647

[ref60] Xiao Z. Li W. Moon H. Roell G. W. Chen Y. Tang Y. J. (2023). Generative artificial intelligence GPT-4 accelerates knowledge mining and machine learning for synthetic biology. ACS Synth. Biol. 12, 2973–2982. doi: 10.1021/acssynbio.3c00310, PMID: 37682043

[ref61] Xu G. Fromholt S. E. Chakrabarty P. Zhu F. Liu X. Pace M. C. . (2020). Diversity in Abeta deposit morphology and secondary proteome insolubility across models of Alzheimer-type amyloidosis. Acta Neuropathol. Commun. 8:43. doi: 10.1186/s40478-020-00911-y, PMID: 32252825 PMC7137436

[ref62] Yang H. Wang T. Qian C. Wang H. Yu D. Shi M. . (2025). Gut microbial-derived phenylacetylglutamine accelerates host cellular senescence. Nat Aging 5, 401–418. doi: 10.1038/s43587-024-00795-w, PMID: 39794469

[ref63] Yeoh Y. K. Zuo T. Lui G. C. Zhang F. Liu Q. Li A. Y. . (2021). Gut microbiota composition reflects disease severity and dysfunctional immune responses in patients with COVID-19. Gut 70, 698–706. doi: 10.1136/gutjnl-2020-323020, PMID: 33431578 PMC7804842

[ref64] Zeng X. Li X. Li X. Wei C. Shi C. Hu K. . (2023). Fecal microbiota transplantation from young mice rejuvenates aged hematopoietic stem cells by suppressing inflammation. Blood 141, 1691–1707. doi: 10.1182/blood.2022017514, PMID: 36638348 PMC10646769

[ref65] Zhang Y. Zhang S. Li B. Luo Y. Gong Y. Jin X. . (2022). Gut microbiota dysbiosis promotes age-related atrial fibrillation by lipopolysaccharide and glucose-induced activation of NLRP3-inflammasome. Cardiovasc. Res. 118, 785–797. doi: 10.1093/cvr/cvab114, PMID: 33757127

[ref66] Zhang J. W. Zhang D. Yin H. S. Zhang H. Hong K. Q. Yuan J. P. . (2023). *Fusobacterium nucleatum* promotes esophageal squamous cell carcinoma progression and chemoresistance by enhancing the secretion of chemotherapy-induced senescence-associated secretory phenotype via activation of DNA damage response pathway. Gut Microbes 15:2197836. doi: 10.1080/19490976.2023.2197836, PMID: 37017266 PMC10078122

[ref67] Zuo T. Zhang F. Lui G. C. Y. Yeoh Y. K. Li A. Y. L. Zhan H. . (2020). Alterations in gut microbiota of patients with COVID-19 during time of hospitalization. Gastroenterology 159, 944–955.e8. doi: 10.1053/j.gastro.2020.05.048, PMID: 32442562 PMC7237927

