# Supplementary material for: Global research trends in gut microbiota and cellular senescence: a bibliometric and visual analysis from 2015 to 2025
Source: Front Microbiol. 2025 Aug 6;16:1623875. doi: 10.3389/fmicb.2025.1623875 (PMC12364940; doi:10.3389/fmicb.2025.1623875)
Supplement: Supplementary file 1 [file Supplementary_file_1.docx]

**Global Research Trends of Gut Microbiota and Cellular Senescence: A Bibliometric and Visualization Analysis from 2015 to 2025**

**Appendix 1**

**Search strategy**

（Cell Senescence OR Cell Aging）AND (Gastrointestinal icrobiome OR Gastrointestinal Microbiomes OR Microbiome, Gastrointestinal OR Gut Microbiome OR Gut Microbiomes OR Microbiome, Gut OR Gut Microflora OR Microflora, Gut OR Gut Microbiota OR GutM Microbiotas OR Microbiota, Gut OR Gastrointestinal Flora OR Flora, Gastrointestinal OR Gut Flora OR Flora, Gut OR Gastrointestinal Microbiota OR Gastrointestinal Microbiotas OR Microbiota, Gastrointestinal OR Gastrointestinal Microbial Community OR Gastrointestinal Microbial Communities OR Microbial Community, Gastrointestinal OR Gastrointestinal Microflora OR Microflora, Gastrointestinal OR Gastric Microbiome OR Gastric Microbiomes OR Microbiome, Gastric OR Intestinal Microbiome OR Intestinal Microbiomes OR Microbiome, Intestinal OR Intestinal Microbiota OR Intestinal Microbiotas OR Microbiota, Intestinal OR Intestinal Microflora OR Microflora, Intestinal OR Intestinal Flora OR Flora, Intestinal OR Enteric Bacteria OR Bacteria, Enteric ）
